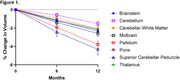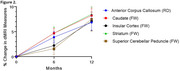# Advanced MRI biomarkers for efficient clinical trial designs in Progressive Supranuclear Palsy (PSP)

**DOI:** 10.1002/alz70856_102985

**Published:** 2025-12-26

**Authors:** Simone P Zehntner, Jean‐Philippe Coutu, Felix Carbonell, Alex P Zijdenbos, Barry J Bedell

**Affiliations:** ^1^ Biospective Inc., Montreal, QC, Canada; ^2^ Biospective Inc, Montreal, QC, Canada

## Abstract

**Background:**

Progressive Supranuclear Palsy (PSP) is a rare neurodegenerative disease characterized by movement, balance, speech, and eye movement difficulties. PSP is a form of four‐repeat tauopathy, involving the abnormal accumulation of tau in the brain. This accumulation leads to neuronal loss, particularly in the brainstem. Identifying robust biomarkers for disease progression is critical for minimizing the sample size in clinical trials. Advanced MRI‐based methods, such as regional volumetric and diffusion MRI (dMRI) processing and analyses, offer promising tools to detect subtle changes in brain structure. These methods allow for the analysis of both regional brain atrophy and microstructural changes, enabling insights into disease progression as well as enable the rapid evaluation of novel therapeutics in clinical trials.

**Methods:**

MRI data from PSP patients and healthy controls were acquired from the 4‐Repeat Tau Neuroimaging Initiative (4RTNI) and Frontotemporal Lobar Degeneration Neuroimaging Initiative (FTLDNI). Fully‐automated image processing was performed using the PIANO™ software platform. Regional analysis focused on brain regions with significant atrophy or microstructural changes. Sample size calculations for clinical trials were performed to estimate the number of participants needed to detect a reduction in observed changes.

**Results:**

Over 12 months, PSP patients exhibited up to 4% volume loss in key brain regions, such as the superior cerebellar peduncles, pallidum, midbrain, and brainstem (Figure 1), and statistically significant microstructural changes in RD and FW in white and gray matter regions (Figure 2). Sample size estimates revealed that fewer than 50 participants per arm were required to detect 60% slowing of changes in the most affected regions. In comparison, alternative methods, such as FreeSurfer and MRPI, required significantly larger sample sizes. The analysis also demonstrated 5‐10% increases in dMRI metrics over 12 months in PSP patients, compared to smaller changes in controls.

**Conclusion:**

The integration of advanced MRI processing techniques, including volumetric and dMRI analyses, enhances sensitivity to brain changes in PSP, significantly reducing sample size requirements for clinical trials. These findings underscore the importance of selecting robust imaging biomarkers and analysis methods to improve trial efficiency and accelerate therapeutic development for PSP.